# Influence of Biofuel Blending on Inorganic Constituent Behavior and Impact in Fluidized-Bed Gasification

**DOI:** 10.1021/acs.energyfuels.4c05818

**Published:** 2025-02-13

**Authors:** Florian Lebendig, Michael Müller

**Affiliations:** Institute of Energy Materials and Devices (IMD-1), Forschungszentrum Jülich, Wilhelm-Johnen-Straße, 52428 Jülich, Germany

## Abstract

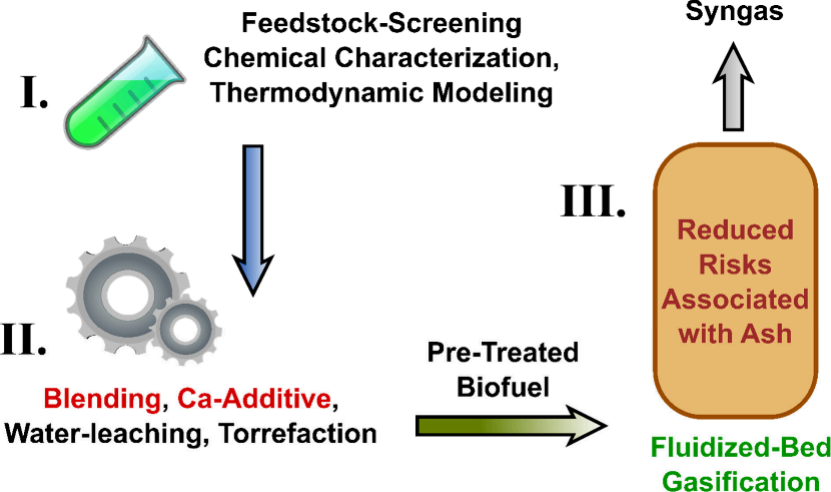

A promising technology for producing carbon-neutral fuels is fluidized-bed gasification of biomass. In combination with chemical looping gasification (CLG), the process becomes even more efficient. However, using biomass-based fuels can lead to significant ash-related issues, including bed agglomeration, fouling, deposition, slagging, and high-temperature corrosion. To address these issues, several biomass upgrading approaches are used to improve the quality of the feedstock for gasification. These approaches include torrefaction, water leaching, and blending with different additives. This study focuses on the influence of additives and biomass co-blending with low-cost biofuels on the behavior of inorganic constituents and under gasification-like conditions at 950 °C and the corresponding impact in fluidized-bed gasification. For example, blending (upgraded) barley straw with 2 wt % CaCO_3_ resulted in a decrease in slag and a corresponding increase in the proportion of solid oxides. This indicates that thermal stability can be expected at operating temperatures up to 950 °C. Similarly, adding Ca/Si-rich biowaste components increases the ash softening point of herbaceous biofuels. Furthermore, the results show that adding Ca-based or woody biofuel components has a chemical effect on the fate of volatile inorganics. For example, increasing the concentration of calcium in the fuel significantly reduces the release of HCl and partially reduces the release of sulfur species, thus reducing the corrosion risk. These results contribute to the development of more efficient and cleaner biomass gasification processes for producing carbon-neutral fuels.

## 1 Introduction

The global energy demand is consistently increasing due to population growth, and it continues to rely predominantly on fossil fuels. The Paris Agreement emphasizes the need to limit global warming to below 2 °C, preferably 1.5 °C.^1^ As a response, there is a gradual shift toward renewable energy, aiming to replace fossil fuels, accounting for approximately 45% of total CO_2_ emissions as of 2020.^2^ In comparison to other renewable sources like wind, solar, or hydroelectric power, biomass holds great promise due to its topographical independence and abundant availability.^3^

Among other fluidized-bed gasification technologies, chemical looping gasification (CLG) is recognized as a promising technology for converting biomass into synthetic fuels.^4^ A detailed description of the multistep process can be found elsewhere.^5^ CLG is achieved through the use of two interconnected fluidized-bed reactors to improve heat and mass transfer efficiency.^6−8^ Various types of fuels can be gasified in the fuel reactor by utilizing a metal oxide (Me_*x*_O_*y*_) as the oxygen carrier. The metal oxide is reduced (Me_*x*_O_*y*–1_) during gasification in the fuel reactor and then moved to the air reactor, where it is oxidized back to its original state using air.

During the gasification process, various challenges arise from biomass ash, including interactions between oxygen carriers and inorganic trace elements, when the system is combined with chemical looping technology, as well as agglomeration and fouling in the fuel reactor, which is relevant for all types of fluidized-bed systems. These issues can lead to operational difficulties and damage to plant components. Therefore, a thorough understanding of ash chemistry is essential to minimize the risks associated with ash. Biofuel upgrading can be employed to mitigate these risks. Although ash-related issues in biomass gasification systems are generally assumed to be similar to those in combustion processes,^9^ the explicit behavior of new biomass fuels under gasification conditions is not well-defined. Hence, a comprehensive fuel characterization focusing on gasification-related properties becomes crucial for their integration.

Torrefaction is a viable method for reducing transportation costs and producing feedstocks with desirable physical properties, such as increased bulk density. Additionally, torrefaction has been found to significantly decrease the amount of chlorine in feedstocks,^10^ resulting in lower levels of alkali chlorides. Water-leaching treatment is an effective method for removing alkali metal salts, including chlorides, carbonates, and sulfates due to their high solubility. In a previous study conducted by Meesters et al.,^11^ extraction experiments demonstrated that four consecutive water extraction steps reduced chloride and potassium concentrations by approximately 80 and 90%, respectively, bringing them within acceptable limits or close to them.

Carefully selecting additives for biomass or fuel blending with low-cost biowaste materials, such as woody feedstocks, offers a promising approach to modify the chemical composition and physical properties of the biofuel, thereby significantly mitigating ash-related risks. This can enable control of the ash melting behavior and release behavior of inorganic species under gasification conditions. The coal industry has successfully employed blending techniques to meet emission targets and minimize ash production during power generation,^12−14^ thereby affecting feedstock quality. Feedstock blending is a widely recognized and effective approach to address ash-related challenges, which vary significantly due to compositional variations.^15,16^ In terms of ash melting behavior, calcium (Ca) and magnesium (Mg) are known to increase the ash melting temperature, while sodium (Na) and potassium (K) decrease it.^17,18^ Additionally, low-melting alkali- and alumina silicates and chlorides can significantly decrease the ash melting point.^16^ Previous studies have demonstrated that the addition of oxides or inorganic salts to the initial biomass can enhance its melting behavior and alter its thermal characteristics.^19−21^

On the basis of the understanding of the CaO–K_2_O–SiO_2_ system, knowledge about fusibility tendencies can be utilized to predict and optimize feedstock quality.^22^ Through blending, the ratio of problematic constituents can be adjusted, thereby improving the overall quality of the feedstock.

Ray et al.^23^ have highlighted the potential of blending and densification processes to enable more cost-effective downstream processing. By utilizing multiple biomass types through blending, the overall land base available for biomass production can be increased, leading to lower total costs for biorefinery feedstock and shorter transportation distances.^24^ Recent studies have also emphasized the opportunity to deliver satisfactory feedstock for biorefineries at lower costs by blending high-quality feedstocks with marginal-quality feedstocks.^25−27^

However, lignocellulosic biomass feedstocks are diverse, making it challenging to define a general pretreatment process applicable to all of them.^28^ While many research studies have focused on analyzing fuel properties through washing, thermal treatment, or torrefaction,^29−31^ there are limited studies available on the combination of these methods with additive blends.^32^ It is crucial to thoroughly investigate and develop the use of blended, densified, and water-leached feedstocks in thermochemical conversion processes, instead of conventionally ground biomass from a single source.

In this study, various investigation techniques have been employed to understand the influence of blending and additivation on the bahavior of inorganics. Molecular beam mass spectrometry (MBMS) was utilized to investigate the release and fate of inorganic volatile species under gasification-like conditions at 950 °C. Hot stage microscopy (HSM) was used to examine the ash fusion characteristics. Lab-scale experiments were complemented with thermodynamic modeling using FactSage to predict the release behavior and mineral phase formations in the ash. On the basis of the findings, specific conclusions regarding the risks of bed agglomeration, slagging, fouling, and high-temperature corrosion in a fluidized gasifier operated at 950 °C were drawn.

## 2 Materials and Methods

### 2.1 Materials and Biofuel Upgrading

The herbaceous feedstocks wheat, barley, corn, and colza straw were purchased commercially from Futtermittel Louven, Germany. The effect of different pretreatment methods, such as torrefaction and water leaching, on the behavior of inorganics of these feedstocks was already investigated in a previous study.^33^ In the present work, they were further improved by blending them with either calcium carbonate or woody biofuel components. Using FactSage, preliminary model calculations were conducted to create various sample blends with oak bark (OB), pine bark (PB), or pine forest residue (PFR). In addition to these woody blend components, ground limestone (CaCO_3_) was selected as a calcium-based additive. It is important to note that the weight ratio between the additive and the pretreated fuel itself, not the ash, is considered for both the woody components and the Ca additive.

In the present study, straw pretreated like in our previous study^33^ was used. The samples underwent a two-step washing process, where each cycle lasted 1 h. The sample–water mixture, comprising 50 g of biomass and 0.5 L of deionized water, was continuously mixed throughout the washing process. Furthermore, samples were torrefied in a pure argon 5.2 atmosphere at a temperature of 250 °C for a duration of 1 h.

Three sample series were created to study the effects of different fuel blending approaches. First, the impact of adding limestone (commercial quality) as an additive to pretreated barley straw was investigated. Second, the effect of different proportions of PB (10–90 wt %) as a co-blending component with colza straw was investigated. The final sample series focused on blending various pretreated fuels with woody components. These blends were chosen based on promising results from preliminary equilibrium calculations. These calculations involved predicting ternary phase diagrams (CaO–K_2_O–SiO_2_) to estimate the solid ash composition of each sample blend at a temperature of 950 °C. The temperature was set at 950 °C to ensure comparability with previous research studies,^32−34^ which were related to CLG utilizing the same unblended biofuels.

### 2.2 Chemical Characterization

The samples were analyzed on their elemental composition with a CHNS analyzer and optical emission spectroscopy combined with inductively coupled plasma (ICP–OES) for the major ash forming elements. Microwave acid digestion of the fuels was applied prior to the determination by ICP–OES,^32^.^33^ Each fuel sample was milled and sieved to a diameter of 0.86 mm to enhance analytical investigations in subsequent steps.

### 2.3 Experimental Hot Gas Analysis by MBMS

The inorganic gaseous species released during gasification were determined in real time using MBMS. A detailed description of the experimental setup can be found in previous publications.^35,36^

Gasification experiments were conducted under gasification-like conditions at a temperature of 950 °C. A four-zone furnace was utilized, with a corundum tube inside the furnace directly connected to the MBMS inlet nozzle. The gasification of the sample occurred in the first two zones at 950 °C. A temperature zone of 1400 °C was set to crack any formed hydrocarbons, ensuring that only inorganic species were investigated.

A total of three measurements were performed for each sample, and the results were averaged for semi-quantitative analysis and error calculations. Similar atmospheric conditions as the ashing procedure were maintained throughout the measurement campaign, which included 15 vol % H_2_O steam and 5 vol % CO_2_ in helium. The total gas flow was set to 4 L/min for each experiment. A 50 mg fuel sample was gasified in a single run. The samples remained in the furnace for varying retention times, typically ranging from 2 min to a maximum of 6 min, based on an initial overview of all spectra. The retention time reflected the reaction sequence, as different species exhibit distinct release behaviors, specifically devolatilization, and subsequent char gasification and ash reactions. Intensity–time profiles of ^23^CO_2_^+2^, ^34^H_2_S^+^, ^35^Cl^+^, ^36^HCl^+^, ^37^Cl^+^, ^38^HCl^+^, ^39^K^+^, ^47^PO^+^, ^55^KO^+^, ^58^NaCl^+^, ^60^COS^+^, ^62^P_2_^+^, ^63^PO_2_^+^, ^64^SO_2_^+^, ^74^KCl^+^, ^81^Na_2_Cl^+^, ^97^NaKCl^+^, ^113^K_2_Cl^+^, ^126^P_2_O_4_^+^, and ^142^P_2_O_5_^+^ were recorded and normalized to the ^23^CO_2_^+2^ base level signal for quantification.

### 2.4 Ash Fusion Testing

The ash of each sample was generated under gasification-like conditions at a constant temperature of 550 °C. The gasifying medium consisted of 15 vol % H_2_O steam in N_2_ and 5 vol % CO_2_. At the start of the process, a small amount of oxygen was introduced to facilitate carbon conversion. A lambda sensor was used to control the partial pressure of oxygen throughout the ashing procedure. When it increased, the oxygen supply was stopped to prevent combustion. Afterward, the ash samples were annealed at 550 °C for 3 h in argon–hydrogen (4% H_2_ in Ar) to ensure the formation of crystalline compounds. For the determination of ash melting behavior using HSM, the ash was pressed into a cylindrical pellet with a diameter of 5 mm, achieving a strength of approximately 1.5 kN. A drop of pure isopropanol was added as a surfactant to maintain stability of the pellet during the pressing process. The height of the sample pellet varied between 4 and 7 mm. The same amount of ash was weighed for each sample preparation. The evaluation was based on the ratio of current sample height/original sample height (coefficient *h*_*x*_/*h*_0_) according to Pang et al.^37^

### 2.5 Thermodynamic Equilibrium Calculations

Thermodynamic equilibrium calculations using the computational package FactSage 7.3^38^ were conducted to predict the formation of inorganic mineral phases of ash constituents under gasification conditions. The commercial database SGPS and the GTOX database^39^ were used for this study. Thermodynamic equilibrium calculations were conducted considering the chemical compositions of the corresponding fuel ashes presented in Table 1. The phase formations in the ash under gasification conditions were calculated considering the addition of water (steam/feedstock = 0.5 g/g) and oxygen (Fe_2_O_3_/feedstock = 0.48 g/g; only oxygen of the oxygen carrier Fe_2_O_3_ was taken into account to exclude possible reactions between oxygen carrier and ash, which were not in the focus of the present investigation).

**Table 1:** Ultimate Analysis of the Fuel Blend Sample Series in wt % or mg kg^–1^ a

Upgraded Barley Straw Blended with 2 wt % CaCO_3_					
	untreated (reference)	torrefied	water-leached	torrefied, post-washed	pre-washed, torrefied
C (wt %)	43.5	50.5	44.5	47.3	49.7
H (wt %)	6.20	5.91	6.37	5.85	5.95
N (wt %)	0.56	0.73	0.39	0.37	0.63
O (wt %)	44.8	38.7	45.7	40.7	41.2
S (wt %)	0.13	0.12	0.10	0.10	0.10
Ash-Forming Components (Major Elements Only)					
Cl (mg kg^–1^)	618	1076	42	60	100
Al (mg kg^–1^)	20	20	20	40	20
Ca (fuel) (mg kg^–1^)	3010	4310	2820	4400	4130
Ca (additive) (mg kg^–1^)	8009	8009	8009	8009	8009
Fe (mg kg^–1^)	7	7	7	49	7
K (mg kg^–1^)	15600	19550	3600	7170	4610
Mg (mg kg^–1^)	377	550	271	515	405
Na (mg kg^–1^)	90	140	90	200	90
P (mg kg^–1^)	890	1258	490	980	1010
Si (mg kg^–1^)	7480	10720	7000	10710	10800

Feedstocks for Fuel Blending (T&P, Torrefied and Post-washed; OB, Oak Bark; PB, Pine Bark; and PFR, Pine Forest Residue)								
	untreated colza	T&P barley	T&P corn	T&P colza	T&P wheat	PFR	PB	OB
C (wt %)	41.2	47.1	48.1	47.6	49.3	52.7	42.1	45.5
H (wt %)	6.01	5.85	5.90	5.98	5.83	6.40	5.66	6.22
N (wt %)	0.51	0.37	0.57	0.36	0.47	0.34	0.40	0.43
O (wt %)	44.1	39.7	40.3	42.2	39.4	40.5	38.7	43.4
S (wt %)	0.33	0.10	0.10	0.10	0.10	0.05	0.10	0.10
Ash-Forming Components (Major Elements Only)								
Cl (mg kg^–1^)	8340	60	273	349	298	70	232	63
Al (mg kg^–1^)	31	40	550	40	61	174	830	239
Ca (mg kg^–1^)	8430	4400	3110	4760	2640	3390	7600	15560
Fe (mg kg^–1^)	7	49	270	18	76	134	830	158
K (mg kg^–1^)	22370	7170	4470	3250	6400	1370	1840	2810
Mg (mg kg^–1^)	552	515	1740	253	588	453	676	516
Na (mg kg^–1^)	1199	200	100	200	200	34	228	2810
P (mg kg^–1^)	242	980	573	130	210	217	409	368
Si (mg kg^–1^)	98	10710	10800	70	10200	958	9200	3880

Torrefied and Post-washed Straw Blended with Woody Biofuel Component (wt %/wt %)						
	barley/OB 65:35	corn/PB 70:30	corn/PFR 60:40	colza/PB 65:35	wheat/PFR 60:40	wheat/OB 60:40
C (wt %)	46.5	46.3	49.9	45.7	50.7	47.8
H (wt %)	5.98	5.83	6.10	5.87	6.06	5.99
N (wt %)	0.63	0.52	0.48	0.37	0.42	0.45
O (wt %)	41.0	39.8	40.4	41.0	39.8	41.0
S (wt %)	0.10	0.10	0.08	0.10	0.08	0.10
Ash-Forming Components (Major Elements Only)						
Cl (mg kg^–1^)	61	261	192	308	207	204
Al (mg kg^–1^)	110	634	400	317	106	132
Ca (mg kg^–1^)	8306	4457	3222	5754	2940	7808
Fe (mg kg^–1^)	87	438	216	302	99	109
K (mg kg^–1^)	5644	3681	3230	2757	4388	4964
Mg (mg kg^–1^)	515	1421	1225	401	534	559
Na (mg kg^–1^)	1114	138	74	210	134	1244
P (mg kg^–1^)	766	524	431	228	213	273
Si (mg kg^–1^)	8320	10320	6863	3266	6503	7672

Untreated Colza Straw Blended with Pine Bark (wt %/wt %)					
	colza/PB 90:10	colza/PB 75:25	colza/PB 50:50	colza/PB 25:75	colza/PB 10:90
C (wt %)	41.3	41.4	41.7	41.9	42.0
H (wt %)	5.98	5.92	5.84	5.75	5.70
N (wt %)	0.50	0.48	0.46	0.43	0.41
O (wt %)	43.6	42.8	41.4	40.1	39.2
S (wt %)	0.31	0.27	0.22	0.16	0.12
Ash-Forming Components (Major Elements Only)					
Cl (mg kg^–1^)	7529	6313	4286	2259	1043
Al (mg kg^–1^)	111	231	431	630	750
Ca (mg kg^–1^)	8347	8223	8015	7808	7683
Fe (mg kg^–1^)	89	213	419	624	748
K (mg kg^–1^)	20317	17238	12105	6973	3893
Mg (mg kg^–1^)	564	583	614	645	664
Na (mg kg^–1^)	1102	956	714	471	325
P (mg kg^–1^)	259	284	326	367	392
Si (mg kg^–1^)	1008	2374	4649	6925	8290

a Note that the composition of the biofuel blends is determined on the basis of the characterization of the raw or upgraded fuels themselves, as presented in their raw form in refs (32 and 33). All compositions were calculated on a percentage basis. For upgraded barley straw blended with 2 wt % CaCO_3_, only the value for Ca was determined on a molar basis, as 2 wt % CaCO_3_ (100.09 g/mol) corresponds to 0.8 wt % Ca (40.08 g/mol).

## 3 Results and Discussion

### 3.1 Fuel Composition

Table 1 presents the ultimate analyses of the fuel sample blends.

### 3.2 Release of Inorganic Constituents

**Figure 1:**
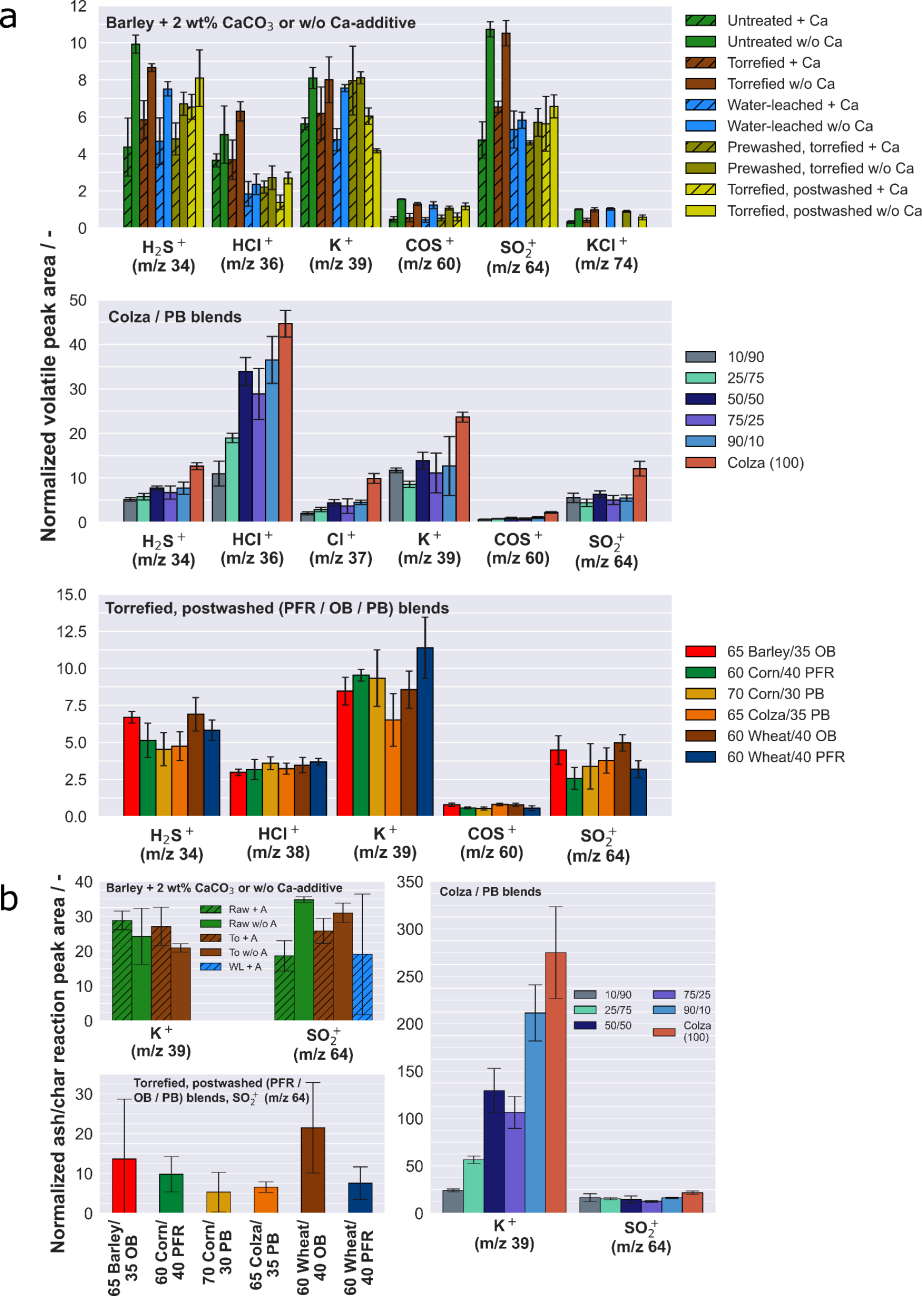
Release of inorganics during gasification-like conditions. (a) Averaged normalized peak areas of detected inorganics during pyrolysis/devolatilization phase at 950 °C. Top, upgraded barley straw blended with and w/o 2 wt % CaCO_3_; center, untreated colza straw co-blended with PB; and bottom, torrefied and post-washed straw varieties, co-blended with varied quantities of woody blends (PFR, OB, or PB). (b) Averaged normalized peak areas of detected species released during the ash/char reaction phase at 950 °C. Note that the non-blended components of biofuel (barley and colza straw), included for comparison, were investigated in ref (33) with permission from the Royal Society of Chemistry.

The release of inorganic compounds was investigated during the pyrolysis/devolatilization phase and ash char reaction phase at 950 °C. Figure 1 shows the semi-quantitative gas phase analysis results for the blended samples under gasification-like conditions. A comparison between upgraded barley straw with 2 wt % CaCO_3_ (Figure 1a) and without the additive, as studied in ref (33), showed differences in the release of inorganic species.

**Figure 2:**
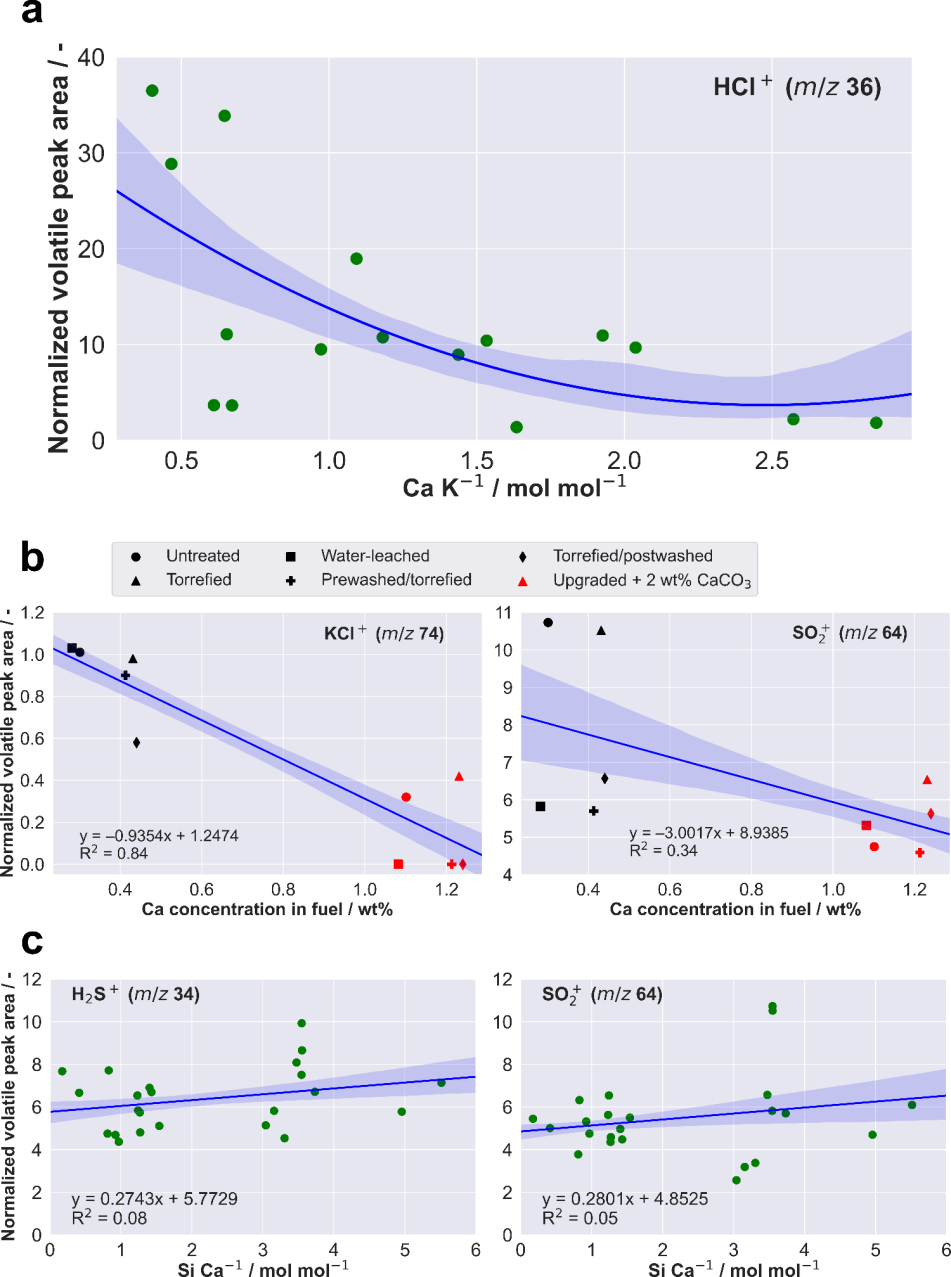
Correlation between the release of inorganic species and fuel composition. (a) Summarized molar Ca/K ratio versus HCl release during devolatilization for all investigated fuel sample series in this study. (b) Correlation between the normalized peak area of KCl and SO_2_ released during pyrolysis phase (upgraded barley straw blended with 2 wt % CaCO_3_ and upgraded barley straw without additive). (c) Summarized molar Si/Ca ratio versus H_2_S and SO_2_ release during devolatilization for the fuel sample series and the corresponding, non-blended reference materials. Note that the non-blended components of biofuel, included for comparison, were investigated in ref (33) with permission from the Royal Society of Chemistry. The confidence interval was set at 68% for panels a, b, and c.

The Ca-blended samples exhibited lower release of sulfur species (H_2_S, SO_2_, and COS) during devolatilization, with a quantifiable decrease of about 50%. It needs to be noted, that due to the short residence time of released species in the reactor before sampling not all initially released SO_2_ is reduced to H_2_S and therefore detectable. Similar observations were found for HCl release, although the decrease was not as pronounced. Figure 2b illustrates the correlated effect of the upgraded and Ca-blended fuels on the release of KCl and SO_2_ during devolatilization.

It is clear that the samples blended with Ca additive exhibited a significant decrease in the release of potassium chloride and sulfur dioxide. The higher the calcium content in the fuel, the lower the release of KCl or SO_2_. Above a calcium content of approximately 1 wt %, hardly any KCl release was observed in the washed, pre- and post-washed samples. It is not surprising that the source material and torrefied sample without Ca additive showed the highest release of SO_2_. Water-leached, post- and pre-washed samples showed the most promising results, with a 50% reduction in SO_2_ release compared to raw or thermally treated material. This effect was further amplified when the upgraded samples were blended with Ca. Additionally, it was observed that Ca had a noticeable influence on the release of KCl. There is likely a cohesive interaction between K, Ca, and S species, which explains the observed mechanisms. A high potassium content in the herbaceous sample leads to retention of sulfur by the formation of potassium sulfate and suppressing SO_2_ emissions. Silica binds potassium, which attenuates the formation of K_2_SO_4_ and promotes SO_2_ release.^40,41^ The presence of calcium in the ash promotes the formation of high-melting calcium silicates, resulting in competition between K and Ca and affecting the release of SO_2_. Ca acts as a S sorbent and modifies the silica network by substituting K, thereby promoting the formation of less harmful potassium sulfate. A study by Wu et al.^42^ reported on sulfates as effective additives for converting KCl to K_2_SO_4_ by destroying KCl. Ultimately, the formation of potassium sulfate is a promising effect, as it inherently restrains the release of both KCl and SO_2_ due to the higher calcium content in the fuel. In summary, Ca exhibits two different chemical mechanisms that effectively suppress sulfur release, and similar findings have been reported for coal/straw blends under combustion conditions.^40^

For the colza samples blended with different proportions of pine bark (PB), it appears that the release behavior of inorganic compounds were mainly influenced by dilution effects. Generally, it can be observed that the sample blend with 10 wt % PB exhibited similar amounts of released inorganic species compared to pure raw colza straw, as referenced in ref (33). In terms of ultimate analysis, raw colza straw had a higher amount of fuel S compared to PB. It can be noticed that the release of S-containing species such as H_2_S, COS, and SO_2_ increased with higher fuel S content. Specifically, the greater the proportion of PB, the lower the quantitative amount of S species released. The same trend was observed for both Cl species, HCl and Cl.

The last series of samples focused on investigating the influence of woody blend components on torrefied and post-washed straw samples. On the basis of the ultimate analyses, the following conclusions can be drawn: the torrefied and post-washed parent straw samples generally had a fuel Ca content ranging from 0.2 to 0.4 wt %, while OB and PB exhibited fuel Ca contents of 1.6 and 0.8 wt %, respectively. The fuel K content was significantly lower for both woody components, averaging 0.2 wt % for woody materials and 0.5 wt % for the upgraded straw sample. The fuel S content remained constant, and the fuel Cl content was approximately the same for the upgraded fuels and the woody components. It is not surprising that fuel Si was generally lower for the woody materials. While OB did not affect the S and Cl species, the quantity of released S and Cl species was partially decreased compared to the upgraded straw material itself. This can likely be attributed to a dilution effect caused by OB.

In Figure 1b, the results of the char gasification/ash reaction, which typically follows the devolatilization phase, are presented. Specifically, for the upgraded barley straw samples with the addition of a Ca additive, only K and SO_2_ were observed to be released. The release behavior during the char gasification/ash reaction did not show any significant differences between the upgraded samples with and without the Ca additive, as mentioned in ref (33). In the samples with the additive, no further release of KCl was observed, which can be attributed to the presence of calcium, as previously discussed.

In the case of the colza/PB blends, the results for the char gasification/ash reaction align with the observations made during the devolatilization phase. A lower proportion of PB blend in colza resulted in a higher release of potassium. This can be attributed to a dilution effect, as raw PB had a lower amount of fuel K compared to colza straw. Additionally, the Si content in the blend is expected to have a significant effect on the release of potassium. PB has a much higher silicon content compared to colza straw, which means it can incorporate more potassium into its networks.

With regard to the release of SO_2_, the ultimate analysis in Table 1 indicated a decrease in the sulfur fraction for sample blends with an increased proportion of PB. The lower fuel S content, therefore, limited the formation of SO_*x*_. Torrefied–post-washed wheat straw, when co-blended with PFR, exhibited a tendency to reduce the release of SO_2_ compared to the OB sample blend. Similar findings were observed during the devolatilization phase.

Figure 2c displays the correlation between the molar fuel Si/Ca ratio and the release of H_2_S or SO_2_. To provide a baseline for comparison, non-blended components from^33^ were also included in the plots. The results reveal a trend, where an increase in fuel Si content is correlated with a higher release of both sulfur species. However, it is worth noting that the inhomogeneity of the biofuels led to the presence of outliers in the data, which, in turn, compromised the accuracy of the results.

On the other hand, an increased fuel Ca content is associated with a reduction in the release of hydrogen sulfide or sulfur dioxide into the gas phase. These findings support the hypothesis regarding the interaction between the Si-network and the Ca cations, which influences the release of S species during devolatilization and gasification.

Lastly, it is important to consider the impact of both Ca and K on the release of HCl. Figure 2a illustrates the relationship between the molar Ca/K ratio and HCl release during devolatilization for the fuel blend materials analyzed. A distinct trend is observed: as the Ca/K ratio increases, the amount of HCl released during devolatilization decreases. These findings align with previous studies on industrially pretreated wheat straw fuels.^32^ In the high-temperature range (950 °C), Ca is primarily present as CaO, a major ash component, and it is possible that CaO acts as an HCl sorbent. Similar observations were reported by Shemwell et al.,^43^ who investigated the treatment of HCl gas with CaO, CaCO_3_, and calcium formate, achieving removal efficiencies of 76, 54, and 81% respectively, depending upon stoichiometry. A recent density functional theory (DFT) study published in 2022^44^ explored the mechanism of HCl capture by CaO and suggested that the oxygen atoms on the surface of CaO play a crucial role in the adsorption of HCl.

It is plausible that a competitive mechanism exists between K and Ca. Thermodynamically, the formation of KCl is more favorable and stable than CaCl_2_. Therefore, CaO can only bind additional HCl if there is no more K available in the gas phase. However, as discussed previously, there is an equilibrium with the silicates. This means that the explanation for this mechanism lies in the Ca/K ratio. However, dependent upon the amount of K relative to Cl, it may not work without Si or may be reduced to Ca + K.

### 3.3 Ash Fusion Behavior

**Figure 3:**
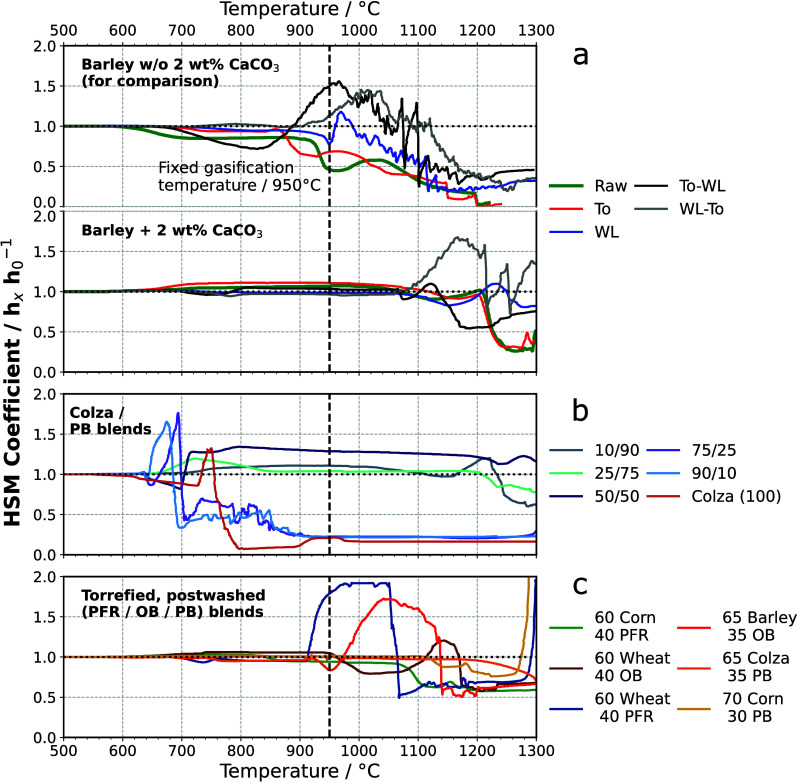
Height profile (coefficient *h*_*x*_/*h*_0_) of ash samples determined by HSM. (a) Upgraded barley straw without and with 2 wt % CaCO_3_. (b) Untreated colza straw blended with varying proportions of PB. (c) Torrefied and post-washed straw varieties blended with woody components. Note that the non-blended components of biofuel (barley and colza straw), included for comparison, were investigated in ref (33) with permission from the Royal Society of Chemistry.

All sample series were analyzed using HSM to investigate ash fusibility characteristics. Note that the fuel blends and their proportions were based on the feedstock itself. Figure 3 illustrates the correlation between the coefficients *h*_*x*_/*h*_0_ of the sample ashes and temperature. Figure 3a presents the ash melting curves for pretreated barley straw without a Ca additive (top panel), with some of the data also presented in ref (33), and for the same material with the addition of 2 wt % CaCO_3_ (bottom panel). It can be observed that the *h*_*x*_/*h*_0_ curves slightly decreased or increased within the low temperature range (600–700 °C). Apart from initial sintering effects, changes in volume may be attributed to residual carbon content in the ash interacting with CO_2_ in the atmosphere. A comparison between the upgraded materials and the Ca-blended samples revealed significant effects on the *h*_*x*_/*h*_0_ curves. The ash from upgraded barley straw without Ca additive displayed noticeable changes above 700 °C (excluding the untreated material itself). Conversely, the upgraded fuels blended with CaCO_3_ demonstrated similar effects from approximately 1050 °C onward. Thermal and leaching pretreatments did not have a significant effect on ash behavior at 1050 °C, and the ash fusion behavior of the Ca-blended fuels remained unchanged. In other words, this suggests that only the presence of the Ca additive had a decisive impact on the ash melting behavior by promoting the formation of high-melting calcium silicates, independent of the upgrading method. In addition, the Ca additive appears to have a more significant impact on the ash fusion temperature compared to thermal and water-leaching treatment.

Figure 3b illustrates the *h*_*x*_/*h*_0_ curves for raw colza straw samples blended with different proportions of PB. Interestingly, the sample blends showed similar ash fusion characteristics as observed for the raw colza straw ash, with ash melting occurring from a temperature range of 630 °C onward. However, for the colza straw sample blended with 90 wt % PB, no effects were observed below 700 °C. Blends with a fraction of 75 wt % PB indicated noticeable effects below 650 °C, and samples with a fraction of either 25 or 10 wt % PB were completely molten at 700 °C. It is not surprising that colza straw ash samples had relatively low melting points, considering that the source material itself had the highest fuel K content and the lowest silicon fraction among all herbaceous biofuels investigated in ref (33). In contrast, PB had a relatively low amount of fuel K and a significantly higher silicon content compared to raw colza straw. On the basis of this fact, it can be reasonably assumed that dilution effects played a key role in the observed ash fusion characteristics. These findings indicate that simply blending K-rich feedstocks with woody components was not sufficient to positively affect the ash melting point. In comparison of torrefied and post-washed colza straw blended to 35 wt % PB, it can be seen that the ash melting point was significantly increased. Because raw colza straw had a relatively low fraction of fuel Si, high-melting silicates could not be formed regardless of whether the fuel Ca content was significantly increased or not. Considering the relatively high fuel Ca content in raw colza straw, this further supports the evidence that high-melting calcium silicates cannot be formed without Si. Thus, this case highlights the difference between herbaceous feedstocks and emphasizes the need for individual upgrading approaches to influence ash chemistry accordingly. In such cases, the melting point could be successfully increased by adding biomass-based silicon-containing additives or blending components with a distinct silicon content.

Lastly, Figure 3c displays the *h*_*x*_/*h*_0_ curves for torrefied and post-washed samples, which were further blended with woody feedstock components. A comparison with the upgraded fuel samples suggested that blending resulted in an increase in the ash melting point for the different sample varieties. For the upgraded fuels, ash melting was observed from temperatures above 700 °C, while the upgraded and co-blended samples exhibited an average melting temperature of 900 °C. The most promising results were obtained for corn straw blended with 30 wt % PB, while PFR showed the least effective results in terms of increasing the ash melting point. It should be noted that corn/PB exhibited the highest fuel Si fraction among all samples examined, with more than 1 wt %. This suggests that Si could form stable high-melting calcium silicates, which positively influenced the ash fusion temperature of the fuel samples. Additionally, biomass upgrading through post-washing had a significant side effect, as the fuel K content was reduced by an average of half. Thus, the formation of low-melting potassium silicates was reduced, thereby diminishing the competition between Ca and K in terms of silicate formation.

### 3.4 Thermodynamic Modeling of the Mineral Phases’ Formation

**Figure 4:**
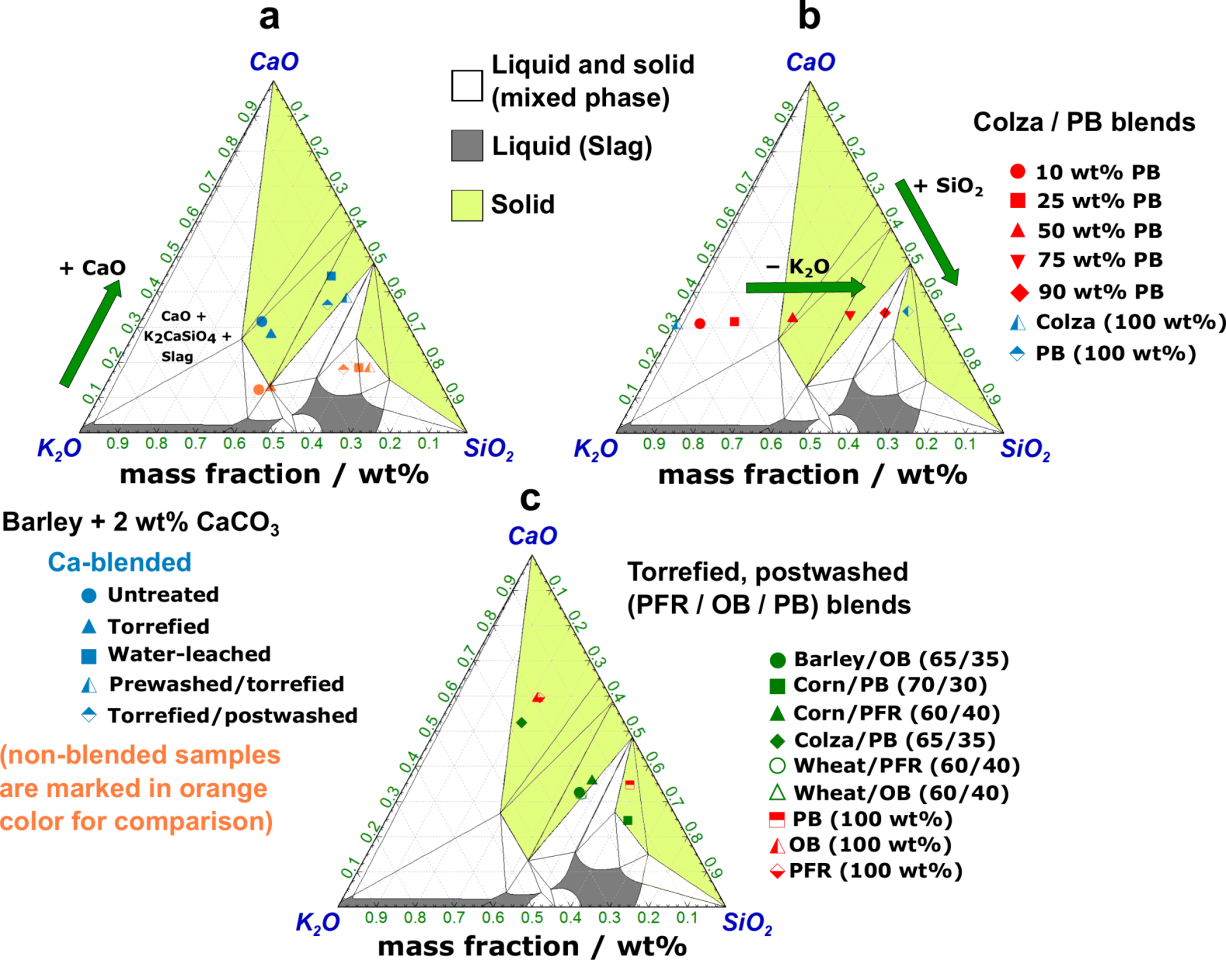
Ternary phase diagrams for the SiO_2_–CaO–K_2_O system at 950 °C and atmospheric pressure, calculated using FactSage.^45^ Predicted phase fields for ashes of (a) pretreated barley straw blended with 2 wt % CaCO_3_, (b) colza straw blended with various proportions of PB, and (c) torrefied–post-washed herbaceous straw varieties blended with various proportions of PB, OB, or PFR. Note that the non-blended biofuels (barley and colza straw), included for comparison, were investigated in ref (33) with permission from the Royal Society of Chemistry, while PFR in its raw form was examined in ref (32).

The ash fusibility of the sample ashes is compared in ternary CaO–K_2_O–SiO_2_ diagrams in Figure 4. Unlike solely upgraded barley straw (Figure 4a), the Ca-blended samples showed distinct effects regarding their ash melting behavior. The green arrow indicates an increase in the fraction of CaO, and it is evident that the Ca additive shifted the sample ash from the two-phase (solid and liquid) region to the solid phase, resulting in solid oxides being the dominant compounds. As a result, no ash melting should occur at the operating temperature of 950 °C. HSM investigations, as shown in Figure 3a, confirmed the stability of the sample ashes at 950 °C.

Colza straw was mixed with different ratios of pine bark in its raw form, and a clear relationship between the blend proportions and the phase composition was observed (Figure 4b). When colza straw had a proportion of 50–75 wt % pine bark, the ash was found to be in the solid phase at the temperature of 950 °C, mainly due to the presence of the dominant oxide phase. Therefore, no ash melting should be expected. It appeared that this effect was primarily due to dilution effects, and the arrows in the diagram indicate a decrease in K_2_O and a corresponding increase in SiO_2_ with an increasing proportion of PB.

The last series of samples (Figure 4c) focused on torrefied–post-washed straw varieties that were blended with woody components. It is worth noting that both woody components, OB and PFR, exhibited nearly identical phase compositions at 950 °C. In contrast to OB and PFR, PB was predicted to be in the two-phase region. Overall, it can be concluded that the most promising results in terms of increasing the proportion of solid oxides were achieved by blending upgraded barley straw with a Ca additive, indicating that thermal stability can be expected at 950 °C. None of the Ca-blended samples investigated were found in the liquid phase. Furthermore, it appeared that simply blending the source material with Ca was sufficient to shift the ash constituents from the two-phase region to the solid phase, eliminating the need for any prior pretreatment to enhance this effect.

**Figure 5:**
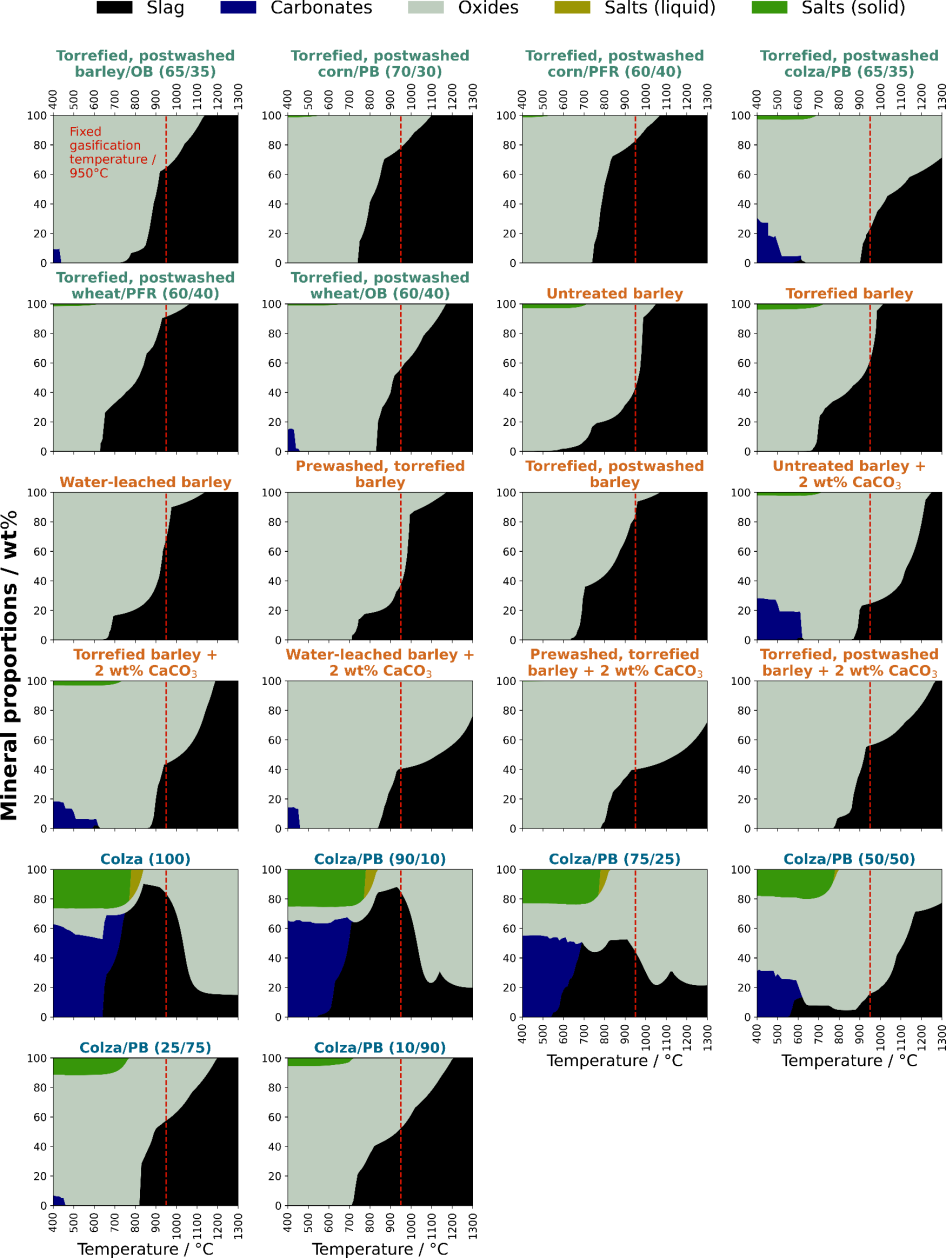
Predicted mineral phase maps under gasification conditions for ashes of (upgraded) straw blends varieties. The dashed line represents the fixed gasification temperature of 950 °C. Note that the non-blended biofuels (barley and colza straw), included for comparison, were investigated in ref (33) with permission from the Royal Society of Chemistry.

Mineral phase maps were predicted for all series of samples and are displayed in Figure 5. It is evident that the ashes from the colza/PB (65:35) and corn/PB (70:30) blends did not exhibit any signs of melting at 950 °C. HSM investigations confirmed the stability of both aforementioned sample blends within the specified temperature range, as no significant changes in sample specimen geometry were observed. However, the ash constituents of the residual sample blends were found to be situated close to the phase boundary between solid oxides and the two-phase region, albeit still in the solid phase. Consequently, they may exhibit thermal instability at 950 °C. On the other hand, these samples demonstrated a relatively high average melt fraction of approximately 75 wt %. Through experimental ash fusion testing using HSM, melting or expanding effects were indeed confirmed.

In contrast to pre-processed barley straw, Ca-blended samples exhibited different phase formations at lower temperatures. Ca promoted carbonate formation, aligning well with the results obtained from the release experiments in Figure 2a. A significant finding was the competition between K and Ca cations. It was hypothesized that Ca modified the silica network, thereby facilitating the formation of high-melting silicates. Consequently, the substitution of K by Ca resulted in an increased fraction of free K remaining in the ash. In their study, Novaković et al.^46^ reported on the release of K from the K–Ca–Si system. They discovered that at elevated temperatures, SiO_2_ selectively reacted with CaO, leading to the release of more K into the gas phase rather than its incorporation into the silicate structure. In line with these findings, it can be suggested that K potentially formed carbonates instead, which could explain the absence of carbonates in the phase maps of pre- or post-washed samples. The carbonate fraction in the solely washed sample was considerably lower than that in the source material or the torrefied sample, indicating that most of the K was leached out during the washing treatment.

With regard to slag formation, Ca-blended barley straw samples have shown promising results. The formation of liquid slag was expected to begin between 800 and 850 °C and appeared to remain consistent up to 950 °C.

In the case of colza straw in its raw form and co-blended with increasing ratios of PB, there was a noticeable decrease in the formation of alkali salts (carbonates and chlorides) at lower temperatures with an increasing fraction of PB. This highlights the relatively high concentration of K in the ash of non-blended colza straw. The transition from solid carbonates to slag occurred smoothly, and based on the predicted thermodynamic activities of the species, the ash–slag mixture predominantly consisted of molten carbonates. It is important to note that both sample ashes, colza/PB (90:10) and colza/PB (75:25), exhibited a significant decrease in molten slag formation from around 950 °C onward. This can be attributed to the increased release of components containing K, resulting in a reduction of slag. As a result, the majority of the formed slag can be attributed to high-temperature stable oxides, such as CaO. In this unique case, it should be mentioned that the investigated colza straw samples had a low silicon content (as seen in ref (33)), therefore no low-melting silicates were expected to form at high temperatures. On the other hand, an increased fraction of PB blend was accompanied by an increased concentration of silicon in the fuel. This led to a higher formation of molten slag when the PB fraction exceeded 25 wt %. Interestingly, even though there were lower amounts of fractional potassium and traces of sodium in the ash, which typically react with SiO_2_ to produce low-melting substances, the ash still progressively fused from 950 °C onward. Consequently, despite the higher proportion of the woody component (≥25 wt %) and the corresponding decrease in fuel K, the potassium content in the fuel was still sufficient to form low-melting silicates at high temperatures.

In summary, the blending ratio of the woody component had significant effects on the phase formations in PB-blended colza straw samples, which were highly dependent upon the ash forming constituents, especially K and Si. Ultimately, the findings clearly demonstrated the crucial role of chemical interactions between the inorganic components in determining the fusibility of the ash.

## 4 Conclusion and Potential Impacts on Fluidized-Bed Gasifier Operation

The study thoroughly investigated the influence of a Ca-based additive and biofuel co-blending on the behavior of inorganic constituents. To predict the ash fusibility at 950 °C, ternary phase diagrams were created based on preliminary thermodynamic calculations. Sample series were then conceptualized. The Ca-based additive and the woody biofuel blending components were found to have significant effects on the formation of gas and mineral phases. The interactions of inorganics were attributed to both chemical effects and dilution.

It is evident that Si, Ca, and K play a crucial role in estimating slagging tendencies, making them fundamental factors for the slagging index. For instance, colza straw ash exhibits a relatively high fraction of KCl but a noticeable low level of fuel Si. In comparison to other herbaceous biofuels, the fraction of molten slag was found to be relatively low in the lower temperature range. Co-blending with woody biofuel components like PB had intriguing effects on ash fusion behavior, including the simultaneous reduction of molten slag formation and increase in the ash melting point. However, an excess of the PB blending component shifted the equilibrium chemistry to favor enhanced molten slag phases at lower gasification temperatures due to an excess of fuel Si derived from the woody component. These findings emphasize the importance of comprehending the overall inorganic fuel composition.

In a recent study,^32^ notable correlations were observed between the Ca content in the fuel and the behavior of KCl release in batch-type experiments. This effect was further highlighted in our current work, as we directly compared Ca-blended barley straw with non-blended feedstock. These findings support the notion that Ca may contribute to the capture and deposition of gaseous alkali chlorides (KCl or NaCl).^47−49^ When the fuel sample was washed before blending with CaCO_3_, there was a significant reduction in KCl release. Furthermore, Ca-blended barley straw exhibited a decrease in the release of sulfur species (such as H_2_S, SO_2_, and COS) into the gas phase compared to non-blended straw samples. The e of sulfuric species was also lower, as they were captured by available Ca. Therefore, the presence of Ca not only improved the ash melting behavior but also had potential technical benefits by chemically binding and preventing the release of KCl or S-containing species to the gas phase. Considering that H_2_S has corrosive effects, the reduced release of H_2_S can be a significant advantage for power plant operations due to its electrochemical nature.

Herbaceous feedstocks typically contain significant concentrations of Cl and S, which results in KCl and K_2_SO_4_ becoming the dominant K-containing compounds.^41,50^ Unlike silicates, especially KCl is relatively volatile. The release of this component can lead to increased deposition on heat transfer surfaces, which in turn reduces heat transfer and increases corrosion rates. Additionally, the release of these species into the gas phase can contribute to the formation of aerosols, such as “submicron” particles.

Furthermore, it was observed that fuels rich in calcium have an impact on the release behavior of HCl. In batch-type release experiments, there was a decrease in HCl release parallel to an increase in the fuel Ca content. When the molar ratio of K was higher than Ca, more HCl was released because most K exists in the form of KCl in herbaceous feedstocks. Given that chlorine can accelerate high-temperature corrosion of reactor equipment during thermochemical conversion,^51,52^ capturing HCl would have a favorable impact on this issue.

Colza/PB blends have demonstrated favorable effects on both their ash fusibility and the behavior of problematic species when the woody component PB comprises 50 wt % or more of the blend. This suggests that using woody-herbaceous fuel blends could be an interesting and cost-effective alternative to expensive chemical additives for controlling ash melting and the release behavior of inorganic compounds. On the basis of these findings, it is possible to produce valuable blends using low-cost fuels, which offers additional economic and ecological benefits by eliminating the need for additional chemical additives.

The removal of alkali species from high-temperature fuel gas is crucial for the smooth operation. Aluminosilicate minerals are commonly used as sorbent materials for alkali removal methods. On the other hand, Ca-based minerals like limestone are primarily used to increase the ash melting point by forming stable Ca silicates with higher melting temperatures. Interestingly, the findings suggest that Ca-based additives also have unintentional positive effects in terms of S/Cl sorption mechanisms, which can have beneficial impacts on gas phase chemistry. Corrosion of reactor components and catalyst poisoning are mainly governed by volatile impurities such as chlorine and sulfur compounds in the gas phase. While the concentration of these impurities can be controlled to some extent through operational conditions and gasification processes, their presence remains an unavoidable issue during fluidized-bed operation. Chemical sorption therefore provides an additional advantage in reducing impurities and harmful pollutant emissions.

The addition of Ca-based additives and various biofuel blends has shown promising results in enhancing the thermal stability of ashes at 950 °C. The slag formation could be significantly reduced when blending barley straw with a Ca additive. Additionally, whether or not thermal or water-leaching treatments were applied beforehand, the additive effectively increased the melting point of barley straw ash, as substantiated by both experimental analysis and thermodynamic modeling. As a result, this suggests that the issues related to slagging, fouling, and bed agglomeration in fluidized-bed gasifiers can be significantly mitigated.

In conclusion, it is important to acknowledge that the thresholds for classifying the quality of biofuels should be determined individually for each type of feedstock. This study clearly highlighted the differences in ash constituents and their concentrations among various herbaceous feedstocks. Therefore, additives or co-blending strategies should be specifically tailored to each feedstock. While predictive methods show promise in predicting ash fusion behavior and mineral phase formation at process temperatures, their application is maybe limited by kinetic constraints. Furthermore, it is worth noting that incorporating small amounts of cost-effective additives or blending with other biofuels is an attractive option for improving the quality of low-grade fuels. This approach allows for the utilization of a wider range of agricultural or biogenic residues. The use of additives and co-blending with woody fuel components has shown significant benefits in affecting both gas and mineral phases.
